# The basic helix-loop-helix transcription factor TCF4 recruits the Mediator Complex to activate gonadal genes and drive ovarian development

**DOI:** 10.1101/2025.02.28.640455

**Published:** 2025-03-04

**Authors:** EV O’Neil, SM Dupont, B Capel

**Affiliations:** Department of Cell Biology, Duke University School of Medicine, Durham NC 27710

**Keywords:** gonad, development, TCF4

## Abstract

The bipotential gonad is the precursor organ to both the ovary and testis and develops as part of the embryonic urogenital system. In mice, gonadogenesis initiates around embryonic day 9.5 (E9.5), when coelomic epithelial (CE) cells overlaying the mesonephric ducts proliferate and acquire the competence to differentiate into the two main cell types of the embryonic gonad, the pre-supporting cells and interstitial cell lineages. While some transcription factors that drive gonadal cell fate are known, HLH factors have not been investigated in this capacity. In the present study, we found that HLH binding sites are highly represented upstream of gonadal genes. We investigated the HLH factor Transcription Factor 4 (TCF4) which is expressed in the CE and GATA4+ somatic cells in both sexes prior to sex determination. TCF4 is maintained in ovarian pre-supporting cells and interstitial cells of both sexes but is silenced specifically in male pre-supporting cells. To characterize TCF4’s role in gonad differentiation *in vivo,* we acquired a mutant mouse model that lacks the TCF4 DNA-binding domain and assessed morphology of the gonads at E15.5. While mutants develop gonads, we observed sex-specific effects on the gonads. Relative to wildtype littermates, SOX9 expression was higher in the Sertoli cells of XY *Tcf4*^*STOP/STOP*^ mutant testes, while FOXL2 and NR2F2 were reduced in the supporting and interstitial cell lineages of XX *Tcf4*^*STOP/STOP*^ mutant ovaries, respectively. Furthermore, the supporting: interstitial cell ratio was altered in XX *Tcf4*^*STOP/STOP*^ ovaries. These effects may occur downstream of changes to epigenetic programming or gene expression in somatic gonadal cells in mutant mice, as TCF4 binds the Mediator complex, RNA polymerase holoenzyme, and chromatin remodelers in early somatic cells. We hypothesize that TCF4 drives a gonadal program that advances female fate but is specifically silenced in male supporting cells as these pathways diverge.

## Introduction

The bipotential gonad is the precursor organ to both the testis and ovary and develops as a part of the urogenital system during embryonic development. In mice, gonad development is initiated around embryonic day 9.5 (E9.5) when cells in the coelomic epithelium (CE) on the ventromedial side of the mesonephros break down the underlying basement membrane, proliferate and begin to accumulate [1, 2]. This thickened layer of cells differentiates into the somatic cell types of the gonad, including both pre-supporting cells and interstitial cells [3–6]. XY supporting cells are the first cell type in the gonad to acquire a sex-specific fate. If a Y-chromosome is present, activation of the *Sry* gene leads to Sertoli cell fate commitment, marked by high expression of *Sox9, Dhh, Fgf9,* and *Amh* [7–10]. Expression of *Sry* must occur during a 6-hr window prior to E11.25 for Sertoli cell commitment, otherwise SOX9 expression is not maintained [11]. In the absence of the *Sry* gene (e.g. in XX embryos), supporting cells commit to granulosa cell fate and express *Wnt4, Foxl2,* and *Rspo1* [12–14]. While the interstitial cell population is transcriptionally distinct as early as E11.5 in both sexes, interstitial cells do not express sex-specific markers until E12.5, likely due to paracrine signals from the male or female differentiated supporting cell lineage [15].

Differentiation of the CE cells into somatic gonadal cells occurs through asymmetric cell division, a process in which unequal segregation of proteins into daughter cells results in distinct cell fates [16]. Segregation of the protein NUMB into a CE daughter cell is necessary for differentiation into a gonad cell lineage, while the daughter cell that does not inherit NUMB is thought to maintain CE cell identity [17]. Thus, NUMB and factors downstream may confer competence to differentiate as gonadal cells. Only cells that have ingressed into the gonad express the gonadal marker *Runx1* and are competent to activate *Sry* [18, 19].

Basic helix-loop-helix (bHLH) transcription factors (TF) are a broad class of transcription factors (TF) that are commonly downstream of the NOTCH/NUMB signaling axis [20]. These proteins are widely expressed and regulate gene expression, cell fate commitment, and other crucial biological processes. On a molecular level, bHLH TFs typically form homodimers or heterodimers and recognize E-box binding domains (CANNTG) [21, 22]. In general, bHLH factors are classified across seven different types based on their function and expression [23]. Of note, the class I bHLH factors, such as MyoD, Twist, and TCF4, are broadly expressed across tissues and are generally associated with transcriptional activation of target genes [24]. Meanwhile, class II bHLH factors, which include SREBP, USF, and TCF21, are more tissue-specific and typically bind with class I bHLH factors to regulate a complement of tissue-specific target genes.

The expression of different combinations of class I bHLH TF specify different somatic cell types in the *C. elegans* gonad, suggesting a crucial role for this class of TFs in gonadogenesis in invertebrate species [25, 26]. Further, bHLH TF regulate differentiation of cells from progenitor populations in many different tissues, including brain and muscle [27–29]. However, while at least a dozen bHLH TFs are expressed early in the somatic cell types of the gonad, their roles in mammalian gonad differentiation are not understood [30, 31]. In the present study, we selected and investigated the role of the bHLH TF transcription factor 4 (TCF4), in murine gonadogenesis. Our results support the working hypothesis that TCF4 recruits the Mediator complex to activate gonadal genes in both sexes. While expression of TCF4 is maintained in interstitial lineages in XX and XY gonads, TCF4 expression becomes sexually dimorphic in the supporting cell lineages: TCF4 is silenced in XY Sertoli cells, whereas TCF4 works with FOXL2 to promote expression of ovarian genes in XX gonads.

## Results

### bHLH binding sites are ubiquitous in up- and down-regulated genes in XY and XX gonads.

**Figure 1.**
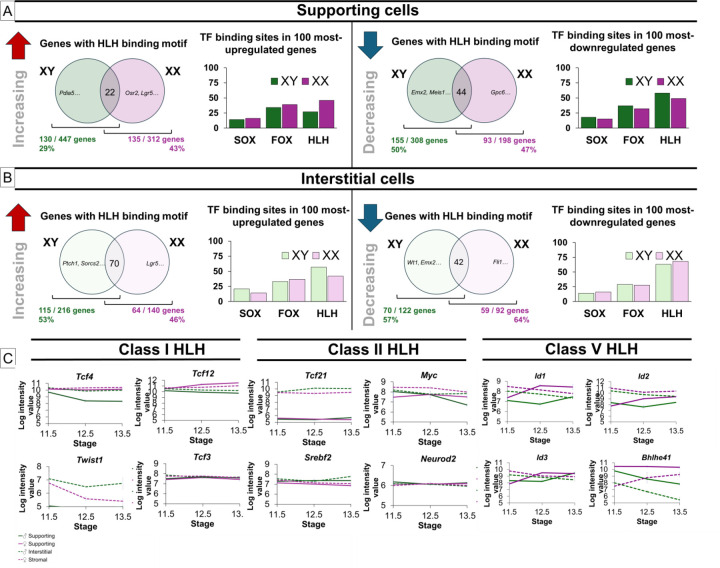
Helix-loop-helix transcription factor binding sites are ubiquitous in dynamically regulated gonadal genes. A) In supporting cells, HLH binding motifs are present in the promoter region of 29% and 43% of genes that increase in XY and XX cells, respectively, and 50% and 47% of genes that decrease in XY and XX cells. Among the top 100 most up- and down-regulated genes in these sets, HLH binding sites are more frequent than SOX or FOX binding sites. B) In interstitial cells, HLH binding sites are present in 53% and 46% of genes that increase in XY and XX cells, respectively. HLH binding sites are enriched in the top 100 most up-regulated genes and occur more frequently than SOX or FOX binding sites. Additionally, HLH binding sites are upstream of 57% and 64% of genes that decrease in XY and XX interstitial cells and are present in nearly 75% of the top 100 most down- regulated interstitial genes. C) Basic helix-loop-helix transcription factors show sex- and cell-type specificity in the gonad (Jameson et al. 2012).

In a screen to identify transcription factor (TF) binding sites upstream of genes expressed in the gonad, bHLH binding sites were found to be over-represented (Garcia-Moreno, unpublished). Investigating this further, we found that bHLH binding sites were more frequent in the promoter region of the top one hundred most increased and decreased genes in both XX and XY supporting cells compared to SOX and FOX TF binding sites, the master regulators of sex determination (Figure 1A). To expand on these findings, we identified genes that were significantly (P < 0.05) up- or down-regulated (1.15-fold) between E11.5 and E13.5 in the supporting and interstitial cell lineages of XY and XX gonads (Jameson et al. 2012) (Supplemental Table 1). In supporting cells, 43% of the genes that were upregulated in XX gonads contained HLH binding sites, compared to 29% of the genes that increased in XY supporting cells (Figure 1A). Meanwhile, HLH binding sites were present in 47% of the genes that were downregulated in XX supporting cells, and in 50% of the genes that decreased in XY supporting cells.

Within the interstitial cell population, HLH binding sites were present in the promoter region of 53% and 46% of increasing genes in XY and XX cells and were once again more common than SOX and FOX binding sites in the top 100 most increased genes (Figure 1B). Additionally, HLH binding sites were present upstream of 57% and 64% of decreasing interstitial genes in the XY and XX gonad, respectively (Figure 1B).

Many HLH transcription factors are expressed within the early gonad (Figure 1C). The Class I HLH factors *Tcf4* and *Tcf12,* which are associated with gene activation, are highly expressed in both supporting and interstitial cells (Figure 1C). Unlike *Tcf12*, *Tcf4* shows a strong down-regulation in the XY supporting cell lineage. The Class II HLH TFs are also associated with gene activation but are either specific to the interstitial lineage (*Tcf21*) or generally expressed at lower levels than the Class I HLH factors. Class V HLH factors, including *Id1, Id2, Id3,* and *Bhlhe41,* are also highly expressed in the gonad but are associated with gene repression. Given the high expression levels and the activating role of TCF4, along with its significant sexually dimorphic expression pattern, we focused on investigating the role of this bHLH factor in early gonad development.

## The bHLH transcription factor 4, TCF4, is highly expressed in the early gonad and down- regulated in SOX9-expressing cells.

**Figure 2.**
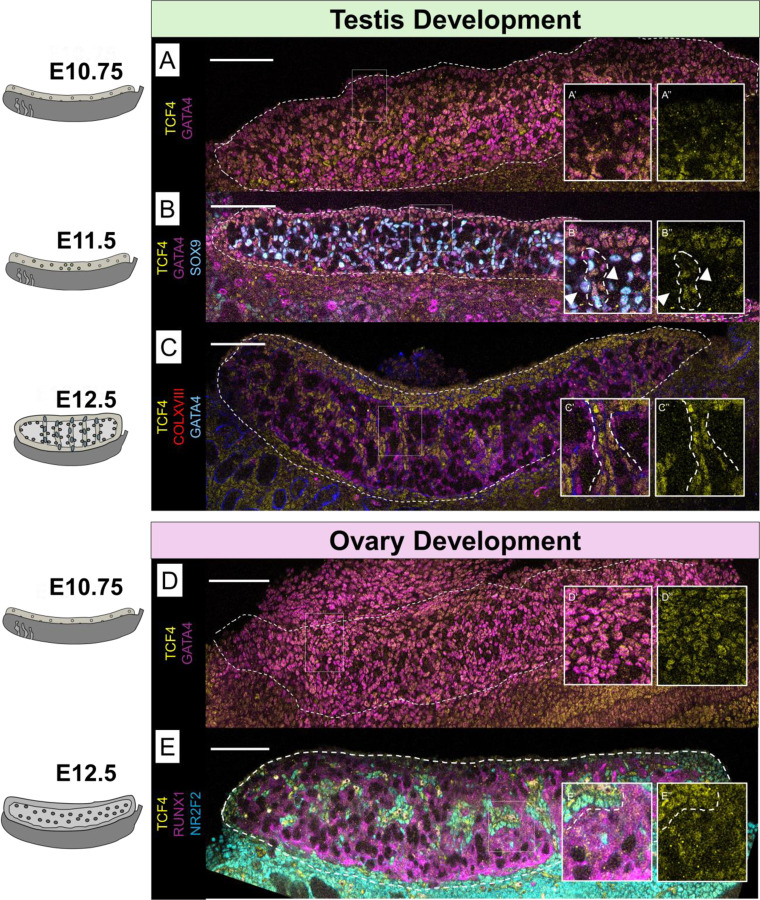
Expression of the HLH transcription factor TCF4 is lost in Sertoli cells but maintained in granulosa and interstitial cell types. A) At E10.75, TCF4 co-localizes with GATA4-expressing gonadal cells in XY gonads. B) At E11.5, TCF4 is lost in SOX9-expressing Sertoli cells in the XY testis, (shown with arrows in B’ and B’’) but is maintained in cells throughout the gonad which are forming sex cords (dotted circle in B’ and B’’). C) In the XY gonad, TCF4 is restricted to the interstitial cell population (shown with dotted lines in C’ and C’’). D) in the XX gonad, TCF4 is co-expressed in GATA4-expressing cells. E) By E12.5, TCF4 is expressed in both RUNX1 expressing granulosa cells and NR2F2+ interstitial cells. Dashed line highlights the boundary between ovarian supporting cells and interstitial cells. Scale bar represents 100 µm.

Using immunostaining (IF), we found that TCF4 was present in the coelomic epithelium and in GATA4+ expressing gonadal cells in XY gonads at E10.75, near the initiation of sex determination (Figure 2A). At E11.5, expression of TCF4 was excluded from SOX9-expressing cells, but was maintained in XY cells that were not SOX9-positive. By E12.5, TCF4 expression became restricted to the interstitial compartment of the testis (Figure 2C), where it co-localized with the interstitial markers, VCAM and NR2F2 (Supplemental Figure 1A). This expression pattern held through E18.5, near the time of birth (Supplemental Figure 1B).

As in the XY gonad, TCF4 was co-expressed in GATA4+ gonadal cells at E10.75 in XX gonads (Figure 2D). However, while TCF4 expression is excluded from Sertoli progenitor cells in the testis, its expression is maintained in both supporting (RUNX1+) and interstitial (NR2F2+) cell types in the developing ovary (Figure 2E). By E18.5, TCF4 is highly enriched in the interstitial lineage, like the testis, but TCF4 maintains colocalization with granulosa cell markers (Supplemental Figure 1C). Collectively, interstitial populations in both ovaries and testes show an enrichment of TCF4 expression, with divergent expression in ovarian and testicular supporting cell lineages.

### Inactivation of the *Tcf4* HLH-DNA binding domain leads to dimorphic effects on the sex determination transcription factors SOX9 and FOXL2.

To understand how loss of T*cf4* affects gonad differentiation *in vivo*, we acquired a knock-out-first mutant mouse line carrying a loxP-P2A-GFP-STOP-loxP cassette in intron 17 of the *Tcf4* gene that produces a truncated form of TCF4 lacking the DNA binding domain [32]. We confirmed the presence of truncated TCF4 by western blot in protein extracts from gonads and limbs of *Tcf4*^*STOP/+*^ and *Tcf4*^*+/+*^ fetuses (Supplemental Figure 2). Homozygous inactivation of *Tcf4* is lethal a few days post-birth, which restricted our analysis of mutants to fetal stages. Mutant *Tcf4*^*STOP/+*^ studs were crossed with *Tcf4*^*STOP/+*^ dams, and *Tcf4*^*+/+*^ and *Tcf4*^*STOP/STOP*^ littermates were collected for downstream analysis.

**Figure 3.**
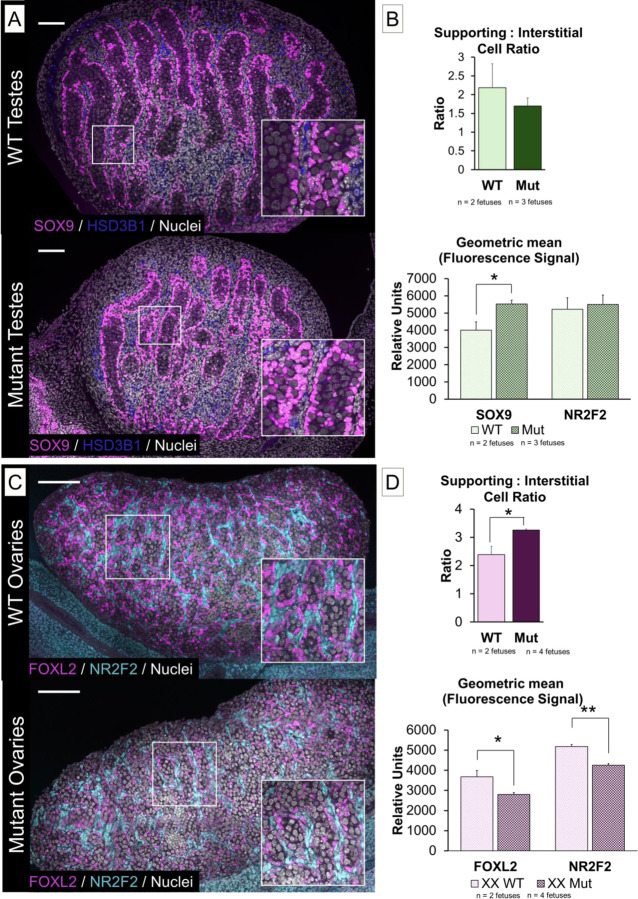
TCF4 does not disrupt gonad differentiation but does affect expression of master regulators of sex determination and cell type ratios in the ovary. A) Immunofluorescence imaging of wildtype *Tcf4*^*+/+*^ and mutant *Tcf4*^*STOP/STOP*^ XY gonads. Representative images shown. B) Gonads were digested and run on a cell analyzer. While ratios of supporting: interstitial cell types did not change, SOX9 was more abundant (P < 0.05) on a per-cell bases in *Tcf4*^*STOP/STOP*^ XY gonads (n=3 gonad pairs) relative to *Tcf4*^*+/+*^ gonads (n=2 gonad pairs). Meanwhile, NR2F2 was not different between samples. C) Immunofluorescence imaging of wildtype *Tcf4*^*+/+*^ and mutant *Tcf4*^*STOP/STOP*^ XX gonads. Representative images shown. B) Gonads were digested and run on a cell analyzer. Mutant gonads had a higher supporting: interstitial cell ratio (P < 0.05). Additionally, FOXL2 and NR2F2 were less abundant (P < 0.05) on a per-cell bases in *Tcf4*^*STOP/STOP*^ XX gonads (n = 4 gonad pairs) relative to *Tcf4*^*+/+*^ gonads (n = 2 gonad pairs).

Both wildtype *Tcf4*^*+/+*^ (n = 3) and mutant *Tcf4*^*STOP/STOP*^ (n = 6) littermates showed relatively normal development of the testis, with organization of germ cells into sex cords surrounded by SOX9+ Sertoli cells (Figure 3A). Additionally, HSD3B1+ interstitial cells were present by E15.5, suggesting that interstitial cell types differentiated normally.

To test whether loss of TCF4 affects lineage specification in the gonads, we measured the proportions of supporting and interstitial cells in E15.5 testes and ovaries. Gonad pairs from mutant (*Tcf4*^*STOP/STOP*^) (n = 3 XY gonad pairs over 3 litters) and wildtype (*Tcf4*^*+/+*^) (n = 2 XY over 2 litters) littermates were separately dissociated, fixed, and stained for supporting cell markers (SOX9 in XY or FOXL2 in XX) and interstitial cell markers (NR2F2). Cells positive for each marker were counted on a Fortessa Cell Analyzer. Gating was set using unstained gonads and single fluorophore positive controls. Supporting cells were identified as positive staining for SOX9 or FOXL2 in XY and XX gonads, respectively, and interstitial cells were identified as positive for NR2F2. The proportions, ratios, and geometric fluorescence values of *Tcf4*^*+/+*^ and *Tcf4*^*STOP/STOP*^ gonads are listed in Supplemental Table 2.

XY *Tcf4*^*STOP/STOP*^ mutant gonads had similar proportions of supporting and interstitial cells relative to wildtype *Tcf4*^*+/+*^ XY gonads. However, there was higher expression of SOX9 protein on a per-cell basis in Sertoli cells of *Tcf4*^*STOP/STOP*^ mutants compared to *Tcf4*^*+/+*^ littermates. There was no difference in levels of the interstitial transcription factor, NR2F2, between wildtype or mutant gonads (Figure 3B).

In XX *Tcf4*^*+/+*^ *(*n = 5) and *Tcf4*^*STOP/STOP*^ (n = 5) fetuses, ovary development also progressed relatively normally, with the appearance and organization of FOXL2+ lined ovigerous cords (Figure 3C). However, some *Tcf4*^*STOP/STOP*^ mutant ovaries had pockets of FOXL2+ granulosa cells lacking germ cells, suggesting that there could be modest disruption to ovarian organization in these mutants (Supplemental Figure 3). Closer analysis of cell types in the ovary on the Fortessa showed that mutant *Tcf4*^*STOP/STOP*^ ovaries (n = 4 gonad pairs over 3 litters) had a greater supporting to interstitial cell ratio relative to XX *Tcf4*^*+/+*^ wildtype (n = 2 gonad pairs over 2 litters) littermates. Further, we observed decreases in the average level of both FOXL2 and NR2F2 (P < 0.05) protein on a per-cell basis relative to wildtype *Tcf4*^*+/+*^gonads (Figure 3D).

### Across both sexes, TCF4 recruits core the Mediator Complex and other core transcriptional machinery to activate gene expression.

**Figure 4.**
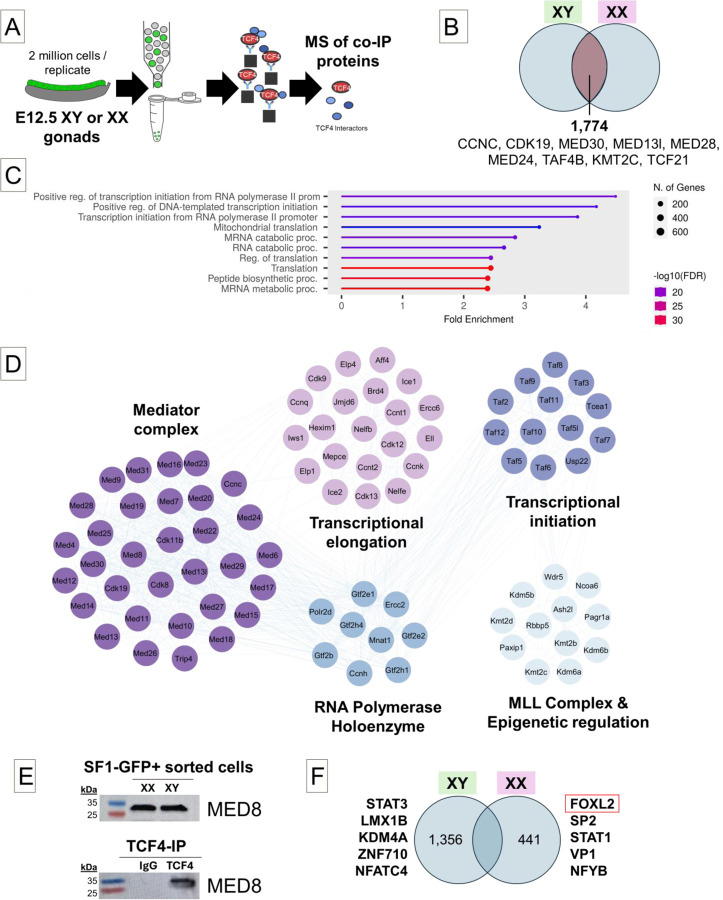
TCF4 binds core transcriptional regulators including the Mediator complex in both XX and XY sorted cells. A) *Nr5Aa1-Gfp* cells were sorted, immunoprecipitated for TCF4, and sent for mass spectrometry analysis to identify TCF4 binding partners. B) In total, 1,774 proteins were identified as TCF4 co-binders common to XY and XX cells. C) Proteins bound by TCF4 were enriched for processes such as positive transcription initiation and regulation of transcription, suggesting that TCF4 is a pro-transcriptional regulator in the gonad. D) Several protein complexes were identified in our mass spectrometry dataset in both XY and XX cells. Notably, the Mediator complex, as well as members of transcriptional elongation, the RNA polymerase holoenzyme, the MLL complex, and transcriptional initiation machinery. Proteins organized in a circle are known complex proteins, and lines indicate interactions. E) XY and XX *Nr5a1-Gfp* cells were sorted and western blot was performed to show the presence of Mediator 8 in somatic gonadal cells (20 µg input). Presence of Med8 was also shown in the western blot of IP’d TCF4 from XY cells (2 million cells / IP). **F**) Additionally, TCF4 co-precipitated with sex-specific proteins, including FOXL2, STAT1, and NFYB in XX cells and STAT3, LMX1B, and KDM4A in XY cells. These proteins were associated with translational machinery in XY cells, and metabolic processing in XX cells.

We were interested in better understanding how inactivation of TCF4 DNA binding culminates in the sex- and cell-type specific effects we observed in the mutant mouse line, so we sought to better characterize TCF4’s actions in the gonad earlier in development. To identify factors that bind with TCF4 in somatic cells of XY and XX gonads, we immunoprecipitated TCF4 from sorted *Nr5a1-GFP* cells at E12.5 and performed mass spectrometry (MS) (Figure 4A) (Supplemental Table 3A, B, and C). NR5A1-GFP can be used to sort both supporting and interstitial cell populations at E12.5 [30, 33]. Binding partners of TCF4 were defined as a 1.5-fold difference between the TCF4 IP and IgG IP in each sex. In total, 2,215 proteins co-precipitated with TCF4 in XX cells, and 3,130 proteins immunoprecipitated in XY cells as defined by a significant enrichment (P = 0.05, rounded to two decimal places) relative to the IgG control IP. Of these, 1,774 proteins co-precipitated with TCF4 in both XX and XY gonads (Figure 4B). TCF4 was enriched in the TCF4 IP of both XX (P = 0.05) and XY cells (P = 0.04). The majority of TCF4 binding partners common to both XX and XY gonads were not differentially abundant by sex (96%) and the HLH factor TCF21, an interstitial-specific bHLH TF, immunoprecipitated with TCF4 in both XX and XY cells. Shared TCF4 binding partners in both XX and XY cells were enriched in factors involved in positive regulation of transcription (Figure 4C). Several key regulators of basal transcriptional processes co-precipitated with TCF4, including all 24 of the Mediator complex proteins, as well as several classes of epigenetic regulators, co-activators, transcription factors, and the RNA polymerase holoenzyme with established roles in transcriptional regulation (Figure 4D). We independently confirmed the presence of one core Mediator protein, Mediator 8, via western blot analysis of a TCF4 pulldown (Figure 4E). While most proteins precipitated with TCF4 in both XY and XX cells, there were also sex-specific TCF4 binders (Figure 4F). Most notably, the core transcription factor, FOXL2, which has well established roles as a crucial regulator of granulosa cell identity, co- precipitated with TCF4 in XX cells.

## Discussion

The sexually dimorphic transcriptional changes that occur during gonad differentiation are well established [30, 31]. However, less is known about upstream factors and protein complexes that drive gonadal fate in cells originally derived from the intermediate mesoderm. In an unbiased investigation of TF binding sites found upstream of some of the earliest regulators of gonad differentiation, including *Nr5a1, Gata4, Wt1,* and *Emx2*, we found that bHLH sites are over-represented [34–37]. Of the many HLH factors expressed in gonadal cells, we chose to investigate TCF4, which is associated with gene activation [38–40], is highly expressed in gonadal somatic cells, and shows a sexually dimorphic pattern with down-regulation specifically in the Sertoli cell lineage.

Recent reports showed that TCF4 is a core transcription factor that binds the Mediator complex to regulate enhancer and chromatin structure in neural stem cells and maintain their progenitor state [40]. To identify the binding partners of TCF4 during gonad differentiation, we performed an IP for TCF4 followed by mass spec. In accord with previous work on TCF4 [40], we identified core transcriptional machinery including the polymerase holoenzyme, epigenetic regulators, the transcriptional co-activators p300/CBP, the TAF4 subunit of general transcription factor II D (TFIID), and every member of the Mediator complex [41, 42].

Sertoli cells are the first cells to differentiate in the gonad. It has been suggested that the window in which they can differentiate is constrained by the advance of a “pan-gonadal” program which drives cells toward ovarian fate [11, 43]. HLH binding sites are found upstream of *Sox9*, where it could recruit activating complexes. SOX9 binds upstream of *Tcf4* [44]. *Tcf4* expression is rapidly down-regulated as Sertoli cells differentiate. In a mutant mouse model where the TCF4 binding domain is disrupted, but the rest of the protein, including the AD domains that recruit p300 and transcriptional machinery, remain intact, we observed elevated expression of SOX9. This finding supports our hypothesis that TCF4 opposes Sertoli fate at the transcriptional level. However, loss of TCF4 does not result in an increase in the proportion of Sertoli cells relative to interstitial cells. In future studies overexpression of TCF4 in SRY/SOX9-expressing Sertoli cells could determine whether TCF4 is inhibitory to Sertoli cell fate.

In contrast to the pattern in the XY gonad, TCF4 is maintained in granulosa cells in the ovary and interstitial cell types of both sexes across gonad differentiation, with highest abundance in the ovarian stroma. We speculate that TCF4 works in conjunction with other TF binding partners such as FOXL2 and TCF21, to specify granulosa and interstitial cell identity, respectively. Of note, Class II HLH factors, such as TCF21, preferentially bind with Class I HLH factors to activate tissue-specific target genes [24, 45]. Thus, TCF4 may be important for TCF21 signaling in the gonad. However, the phenotype of mutant *Tcf4*^*STOP/STOP*^ mice did not match that reported in *Tcf21* mutants, where inactivation of *Tcf21* severely compromises gonad differentiation and leads to hypoplastic testes with increased numbers of interstitial cells [46]. Thus, the exact regulatory mechanism between these HLH factors remains to be deciphered.

In *Tcf4*^*STOP/STOP*^ mutants, we observed decreased expression of the granulosa marker FOXL2 and the ovarian stromal marker, NR2F2 at the single-cell level. Surprisingly, the ratio of granulosa cells to stromal cells is increased in mutants, despite lower levels of FOXL2. This may be explained by the decrease in the stromal population in mutant ovaries, which lowers the denominator and thus increases the granulosa-to-stromal cell ratio. The decrease in FOXL2 and NR2F2 in granulosa and stromal cells is consistent with the hypothesis that TCF4 positively regulates the ovarian pathway.

**Figure 5.**
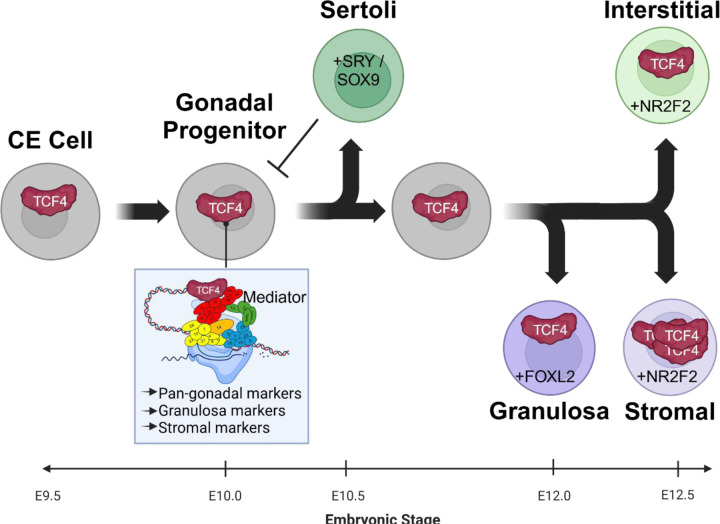
Working model for the role of TCF4 in gonad differentiation. TCF4 drives a gonadal program, likely through recruitment of the Mediator complex and epigenetic factors, that advances ovarian fate and is specifically silenced in Sertoli supporting cells as these pathways diverge. Figure created with Biorender.

In conclusion, we suggest that the TCF4 complex regulates enhancer and chromatin structure to maintain the progenitor state of gonadal cells and regulate expression of granulosa and stromal factors (Figure 5). We hypothesize that *Tcf4* is silenced in Sertoli cells to permit their differentiation but maintained in other lineages where it partners with other proteins such as FOXL2 and TCF21 to drive other gonadal fates. Many other bHLH proteins are expressed in the gonad, and further investigation of this complex is warranted.

## Materials & Methods

### Mouse strains and lines.

All mice were housed in accordance with the National Institutes of Health guidelines, and experimental procedures were approved by the Duke University Medical Center Institutional Animal Care and Use Committee. For wildtype matings, CD-1 mice were obtained from Jackson Laboratories. The Tg(*Nr5a1-GFP)* (*Sf1-eGFP)* mice have been previously described [33]. The *Tcf4*^*STOP/+*^ mutant mouse line was maintained on a C57BL/6J background and was a gift from Ben Philpot at UNC where they have been previously characterized [32, 47].

### Timed matings.

To obtain embryos at specific embryonic stages, females were placed in timed mating cages with studs. Female mice were checked daily for the presence of a vaginal plug, which was considered embryonic day 0.5. Female CD-1 mice were used for all experiments except those involving *Tcf4*^*STOP/+*^ *mice*. For *Tcf4*^*STOP/+*^ matings, *Tcf4*^*STOP/+*^ studs were crossed with *Tcf4*^*STOP/+*^ females.

### Organ collection and genotyping.

Gonad-mesonephric complexes were microdissected out into PBS and used for downstream applications. Tail somites were counted to ensure mice were at the appropriate developmental stage. Tails or limbs were recovered from each fetus and digested in 150 mM NaOH at 95ºC for 15 minutes and neutralized with 1M Tris HCl (pH 7.4) for genotyping. Fetuses were sexed by genotyping for the *Utx* and *Uty* genes using the following three primers (5’→3’): UTX F1: TCATGTCCATCAGGTGATGG, UTY F2: CAATGTGGACCATGACATTG, UTX/Y R: ATGGACACAGACATTGATGG. Mice from the *Tcf4*^*STOP/+*^ breeding scheme were genotyped for the wildtype allele (primer sequences 5’ → 3’ F: GCACTTCAGGGATCGCTTA, R: CCGCCCTAATTGTTCAAAGAG) or transgenic knock-in allele (primer sequences 5’ → 3’: F: GCTGATCCGGAACCCTTAAGC, with the TCF4 WT R primer). PCR conditions have been previously reported [47, 48]. In the case of *Nr5a1-GFP* mice, fluorescent signal was strong enough to observe with a fluorescence detection system on the dissection microscope (e.g. NIGHTSEA), bypassing the need for genotyping.

### Whole mount Immunofluorescence.

Embryonic gonads were fixed for 1 hour at room temperature (RT) or overnight (O/N) at 4ºC in 4% paraformaldehyde while rocking. Samples were rinsed 3 times for 10 minutes in PBS and then passed through an increasing methanol gradient in PBS (25%, 50%, 75%, 100%). Gonads were stored in 100% methanol O/N or longer, as needed. Samples were rehydrated stepwise into PBS and rinsed 3 times in PBS. Late-stage gonads (E15.5) were incubated in 2% Triton X-100 in PBS for 1 hour at RT to increase permeabilization. Early and late-stage gonads were then incubated in blocking buffer (0.1% Triton X-100 for E10.5-E13.5 or 1% Triton X-100 for E15.5 and later, with 3% BSA, and 10% fetal bovine serum or horse serum [HS] in PBS) for 1 hour. Tissue was incubated with primary antibody in blocking solution O/N. Antibody details and concentrations are listed in Table 1. The following day gonads were rinsed in either 0.1% (E10.5-E13.5) or 1% Triton X-100 (E15.5 and later) in PBS and incubated with secondary antibodies (1:500) in blocking buffer O/N. Gonads were rinsed 3 times for 10 minutes in PBS and then mounted in DABCO mounting media. Images were captured with a Leica TCS SP8 confocal microscope using the associated Leica software (Leica, Wetzlar Germany). Images were processed using FIJI (ImageJ).

### Mapping HLH, SOX, FOX, and COUP binding sites.

HLH motif file was generated for the E-box motif (CANNTG) using the “seq2profile” command from HOMER. HLH sites genome wide were identified using the “scanMotifGenomeWide” tool against the mm9 genome [49]. The resulting bed file from this motif scan, as well as bed files from SOX9 and FOXL2 ChIP-seq studies [44, 50], were processed using the tool “annotatePeaks” to identify nearest gene transcription start sites to each genomic location present in the bed files. Next, gene lists unique to XX and XY supporting and interstitial cells were filtered by their fold-change between E11.5 and E13.5. Genes that showed a 1.15-fold change and that were statistically significant (P < 0.05) were defined as ‘Increasing’ or ‘Decreasing’ in the supporting and interstitial genes and output as a csv file (Supplemental Table 1). Annotated bed files were converted to csv and uploaded to R studio alongside the gene lists. Gene lists and motif/ChIP sites were compared for overlap using the function “inner_join” based on Gene Symbol. HLH binding sites were searched for within 1 kilobase of the gene of interest.

### Fluorescence Activated Cell Sorting (FACS) Analysis.

For cell analysis of E15.5 *Tcf4* ^*WT/WT*^ or *Tcf4*^*STOP/STOP*^ gonads, gonad pairs from single fetuses were digested in TrypLE (Gibco, Thermo Fisher, Waltham MA) with 0.1% collagenase for 10 minutes at 37ºC. TrypLE was removed from samples and samples were then resuspended in 3% BSA in PBS and filtered through a 50-um filter. Samples were spun down at 600 g for 10 minutes at 4ºC and then fixed in 4% PFA with 0.1% Triton X-100. After a 10 minute incubation, samples were spun down again at 600 g for 10 minutes at 4ºC. Samples were left in 3% BSA in PBS at 4ºC for up to ten days before primary antibody staining. Cells were recovered, spun down at 600 g for 10 minutes at 4ºC, and then incubated in blocking buffer (10% HS with 0.1% Triton X-100 in PBS) O/N with an antibody to mark interstitial cells (NR2F2) or supporting cells (SOX9/FOXL2 antibody depending on the sex of the sample) (Antibody details in Table 1). The following day samples were spun down at 600 g for 10 minutes at 4ºC and placed in blocking buffer with secondary antibody (Donkey anti-Mouse Cy3, donkey anti-rabbit AF647, or donkey anti-goat AF647) for 1 hour at RT. Samples were spun down and rinsed in 3% BSA in PBS twice before analysis. For FACS analysis, wildtype and mutant gonad suspensions were run on an BD LSRFortessa^™^ Analyzer in a single session. The area of the forward side scatter (FSC-A) and side scatter area (SSC-A) were plotted to isolate cells and remove debris, and the FSC-A and FSC-H were plotted to obtain single cells. Gating conditions were established using unstained gonad cells, and single fluorophore controls (AF657 and Cy3). Voltage settings were as follows: SSC 246, FSC 360, APC 571, PE 505. Next, 5,000 cells from gonad pairs of individual, genotyped *Tcf4*^*WT/WT*^ *and Tcf4*^*STOP/STOP*^ fetuses were recorded. Gating was set at a signal of > 10^2.5^ for the 647 fluorophore and > 10^3.1^ for the Cy3 fluorophore. Cells with fluorescence signal greater than these gating values were identified as ‘supporting’ or ‘interstitial’ cells. The ratio of supporting: interstitial cells was then calculated and compared between wildtype and mutant conditions using a two-tailed t-test. The geometric mean, which gives the average fluorescence on a single-cell level in heterogenous populations, was also determined and compared between wildtype and mutant conditions using a two-tailed t-test.

### Cell sorting.

For cell sorting of *Nr5a1-GFP* gonads at E12.5, fetuses were sexed by PCR, and the gonads were dissected out, pooled by sex, and dissociated for 10 minutes in TrypLE at 37ºC. After digestion, TryPLE was carefully removed, and gonads were resuspended in 10% HS in PBS. The cell suspensions were filtered through a 50-micron filter and sorted on either a BD FACSAria or Astrios Cell Sorter. GFP expression was used to sort somatic cell types, with gating set using a GFP-negative control sample by a Duke Flow Cytometry Core specialist. Cells were recovered, spun down at 3500 g for 10 minutes at 4ºC, snap-frozen, and stored at −80ºC for downstream pooling and IP.

### IP-MS.

Frozen sorted somatic gonadal cells were pooled into three replicates of 4 million cells. Cell pellets were lysed in a Pierce^™^ IP Lysis Buffer (Thermo Fisher) containing 1 mM PMSF and 1x Halt^™^ Protease Inhibitor Cocktail (Thermo Fisher), incubated on ice for 30 minutes, and centrifuged at 14,000 g for 10 minutes at 4ºC. Meanwhile, 50 µl of Protein G Dynabeads (Invitrogen, Waltham MA) were rinsed 3 times in PBS with 0.05% Tween-20 using a magnet bar. Beads were suspended in 237.5 µl of Conjugation buffer (20 m HEPES in PBS), and 5 µg of IgG or TCF4 antibody (Abcam, NCI-R159–6) was crosslinked to the beads using 12.5 µl of 100 mM BS3. Beads were gently vortexed and incubated for 30 minutes in crosslinking buffer. After incubation, beads were resuspended in 250 µl of Conjugation buffer and quenched with 12.5 µl of 1 M Tris HCl (pH 7.5). Beads were incubated for 15 minutes at RT and rinsed 3 times in PBS with 0.05% Tween-20. The extracted protein was then split between IgG or TCF4-conjugated beads and incubated O/N on an end-over-end rotator at 4ºC. The following day beads were rinsed in dilution buffer (10 mM Tris HCl pH 7.5 with 150 mM NaCl and 0.5 mM EDTA), dilution buffer with 1% Triton X-100, dilution buffer with 1% Triton X-100 and 360 mM NaCl, and finally in dilution buffer. Proteins were eluted at 80ºC for 10 minutes in 25 mM Tris, 50 mM NaCl, and 1% SDS and submitted to the Duke Proteomics Core for MS analysis.

### Quantitative LC-MS.

Samples were spiked with 1 or 2 pmol bovine casein as an internal quality control standard. The samples were brought to a final concentration of 5% SDS and reduced for 15 minutes at 80ºC. After reduction, the samples were alkylated with 20 mM iodoacetamide for 30 minutes at RT, then supplemented with 1.2% phosphoric acid and 416 µl of S-Trap (Protifi, Firport NY) binding buffer (90% MeOH/100 mM TEAB). Proteins were trapped on the S-Trap microcartridge, digested with 20 ng/µl of trypsin (Promega, Madison WI) for 1 hour at 47 ºC, and eluted sequentially with 50 mM TEAB, followed by 0.2% formic acid (FA) and 50% aceconitrile/0.2% FA. All samples were lyophilized and resuspended in 1% TFA/2% acetonitrile with 12.5 fmol/µl of yeast alcohol dehydrogenase. A study pool sample was created by combining equal volumes of each sample. Quantitative LC/MS was performed on an EvoSep One UPLC coupled to a Thermo Orbitrap Astral high-resolution, accurate-mass tandem mass spectrometer (Thermo Fisher). Each sample was eluted from the EvoTip onto a 1.5 µm EvoSep 150 µm ID x 1 5cm performance EvoSep column using the SPD30 gradient at 55ºC. Data collection on the Orbitrap Astral mass spectrophotometer was performed in a data-independent acquisition (DIA) mode of acquisition with a resolution of 240,000 for full MS scans in the m/z range of 380–980. An HCD collision energy setting of 27% was used for all MS2 scans.

### Quantitative Proteomics Data Analysis.

Raw data were imported into Spectronaut (Biognosis, Zurich Switzerland) and individual LC-MS data files were aligned based on the accurate mass and retention time of detected precursor and fragment ions. Relative peptide abundance was measured based on MS2 fragment ions of selected ion chromatograms of the aligned features across all runs. The MS/MS data was searched against a SwissProt M. musculus database and an equal number of reversed- sequence “decoys” for false discovery rate determination. A library free Direct DIA+ approach within Spectronaut was used to perform the database searches. Database search parameters included a fixed modification on Cys (carbamidomethyl), with variable modification on Met (oxidation) and Lys (biotin). Full trypsin enzyme rules were applied, along with 10ppm mass tolerances on precursor ions and 20ppm on product ion. Spectral annotation was set at a maximum 1% peptide false discovery rate, based on q-value calculations. Peptide homology was addressed using razor rules, where a peptide that matches to multiple different proteins is exclusively assigned to the protein with the most identified peptides. Protein homology was handled by grouping proteins that had the same set of peptides to account for their identification. Fold-changes were calculated between the IgG and TCF4 group using a two-tailed heteroscedastic t-test on log2-transformed data. The P-Value was rounded to two decimals points. Proteins were enriched TCF4 binding partners if they met the criteria of being at least 1.5-fold higher than the IgG value with a P-value ≤ 0.05.

### Western blot analysis.

Protein was extracted from *Nr5a1-GFP* cells using Pierce^™^ RIPA Lysis Buffer (Thermo Fisher) with 1 mM of PMSF and 1x Halt Protease and Phosphatase inhibitor cocktail (Thermo Fisher). Protein isolates from *Nr5a1-Gfp* cells were quantified using a BCA assay. For the western blot, 20 µg of protein extract or 30 µl of eluant from the IgG or TCF4 IP (approximately half the total eluant from 4 million cell IP reaction split between IgG or TCF4 antibody) was reduced with 5x Laemmli buffer and 1 mM DTT at 95ºC for 5 minutes. Samples were loaded onto a mini-PROTEAN TGX precast gel (Bio-Rad, Hercules, CA) and proteins were separated via SDS-PAGE at a constant voltage of 100 V for 90 minutes in running buffer (25 mM Tris, 192 mM glycine, 0.1% SDS). Protein was transferred using a Trans-Blot Turbo Transfer System (Bio-Rad), following the Manufacturer’s Instructions. Membrane was blocked for 1 hour at 5% milk in 0.1% Tris-Buffered saline with 0.1% Tween-20 (TBST) and incubated O/N with a polyclonal rabbit MED8 or rabbit TCF4 antibody. Blot was rinsed three times in 0.1% TBST and then put in blocking buffer with goat anti-rabbit horseradish peroxidase (HRP) conjugated secondary antibody at a 1:10,000 dilution for 1 hour at RT. Blot was rinsed three times in 0.1% TBST and developed in Super Signal West Pico Chemiluminescent Substrate (Thermo Fisher) for 5 minutes before visualization on an Amersham Imager 600. For non-IP western blots, blots were stripped in Restore PLUS Western Blot Stripping buffer for 15 minutes at RT. Blots were blocked for 1 hour in 5% milk in 0.1% TBST again and then incubated with a polyclonal goat anti-GAPDH antibody (1:5,000) O/N. The following day, the blot was rinsed three times in 0.1% TBST and incubated in a donkey anti-goat horseradish peroxidase (HRP) conjugated secondary antibody. Development and imaging were repeated as previously described.

## Supplementary Material

Supplement 1

Supplement 2

Supplement 3

Supplement 4

## Figures and Tables

**Table 1.** Key Resources.

Reagent type (species) or resource	Designation	Source or reference	Identifiers	Additional information
Gene (*M. musculus*)	Transcription factor 4 (Tcf4)	GenBank	MGI:98506	
Strain, strain background (*Mus musculus*)	Crl:CD1(ICR)	Charles River	Strain code: 022	
Strain, strain background (*M. musculus*)	C57BL/6J	Jackson Laboratory	Stock #:000664	
Genetic reagent (*M. musculus*)	Tg(Nr5a1/EGFP)1Klp	PMID:12351700	MGI:5493455	
Genetic reagent (*M. musculus*)	Tg(Tcf4^STOP/+^)	PMID: 32765228		
Antibody	Smooth muscle alpha actin (aSMA) (FITC-conjugated mouse monoclonal)	Sigma-Aldrich	F3777	1:500
Antibody	Endostatin/Collagen 18 (goat polyclonal)	R&D Systems	AF570	1:500
Antibody	FOXL2 (rabbit polyclonal)	Gift from Dagmar Wilhelm		1:250
Antibody	GATA4 (mouse monoclonal IgG_2α_)	Santa Cruz Biotechnology	Sc-25310	1:250
Antibody	GFP (chicken polyclonal)	Abcam	Ab13970	1:1000
Antibody	MED8 (rabbit polyclonal)	Proteintech	12182-1-AP	
Antibody	NR2F2 (mouse monoclonal IgG_2α_)	Abcam	Ab41859	1:200
Antibody	NR5A2 (goat polyclonal)	Santa Cruz Biotechnology	Sc-21132	1:500
Antibody	SOX9 (goat polyclonal)	R&D Systems	AF3075	1:500
Antibody	TCF4 (rabbit monoclonal)	Abcam	Ab217668	1:500
Antibody	TRA98/GCNA1 (rat monoclonal)	Abcam	Ab82527	1:500
Antibody	AF488 Anti-Mouse (donkey polyclonal)	Jackson ImmunoResearch	715-545-150	1:500
Antibody	AF488 anti-Rat (donkey polyclonal)	Life Technologies	A-21208	1:500
Antibody	AF488 anti-Chicken (donkey polyclonal)	Jackson ImmunoResearch	703-545-155	1:500
Antibody	Cy3 anti-Goat (donkey polyclonal)	Jackson ImmunoResearch	705-165-147	1:500
Antibody	Cy3 anti-Mouse (donkey polyclonal)	Jackson ImmunoResearch	715-165-150	1:500
Antibody	AF647 anti-Rabbit (donkey polyclonal)	Jackson ImmunoResearch	711-605-152	1:500
