# Supplementary figures and images for: The basic helix-loop-helix transcription factor TCF4 recruits the Mediator Complex to activate gonadal genes and drive ovarian development
Source: bioRxiv. 2025 Jul 11:2025.02.28.640455. Originally published 2025 Mar 4. Preprint. [Version 2] doi: 10.1101/2025.02.28.640455 (PMC11908221; doi:10.1101/2025.02.28.640455)

Supplemental Figure 1

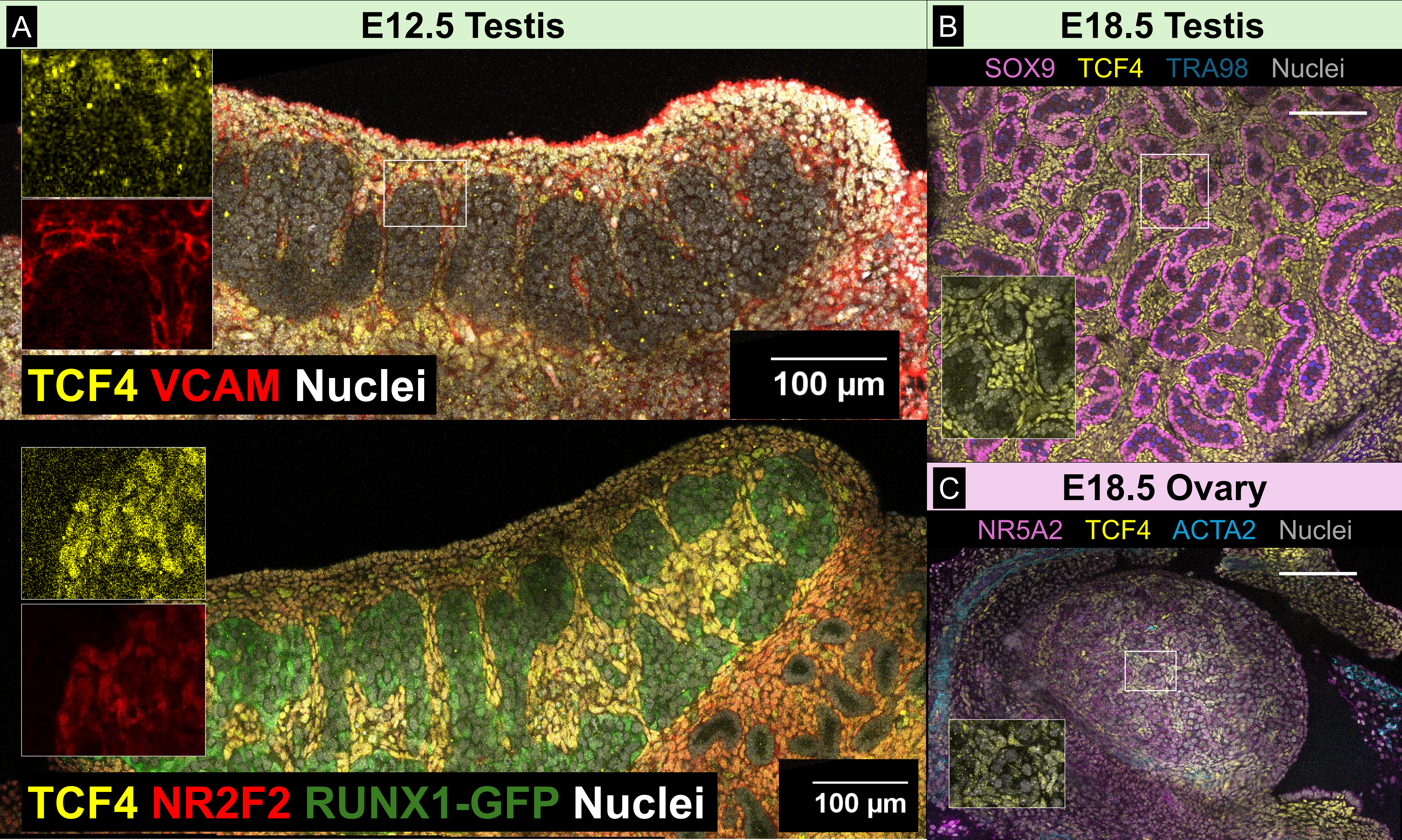

Supplemental Figure 2

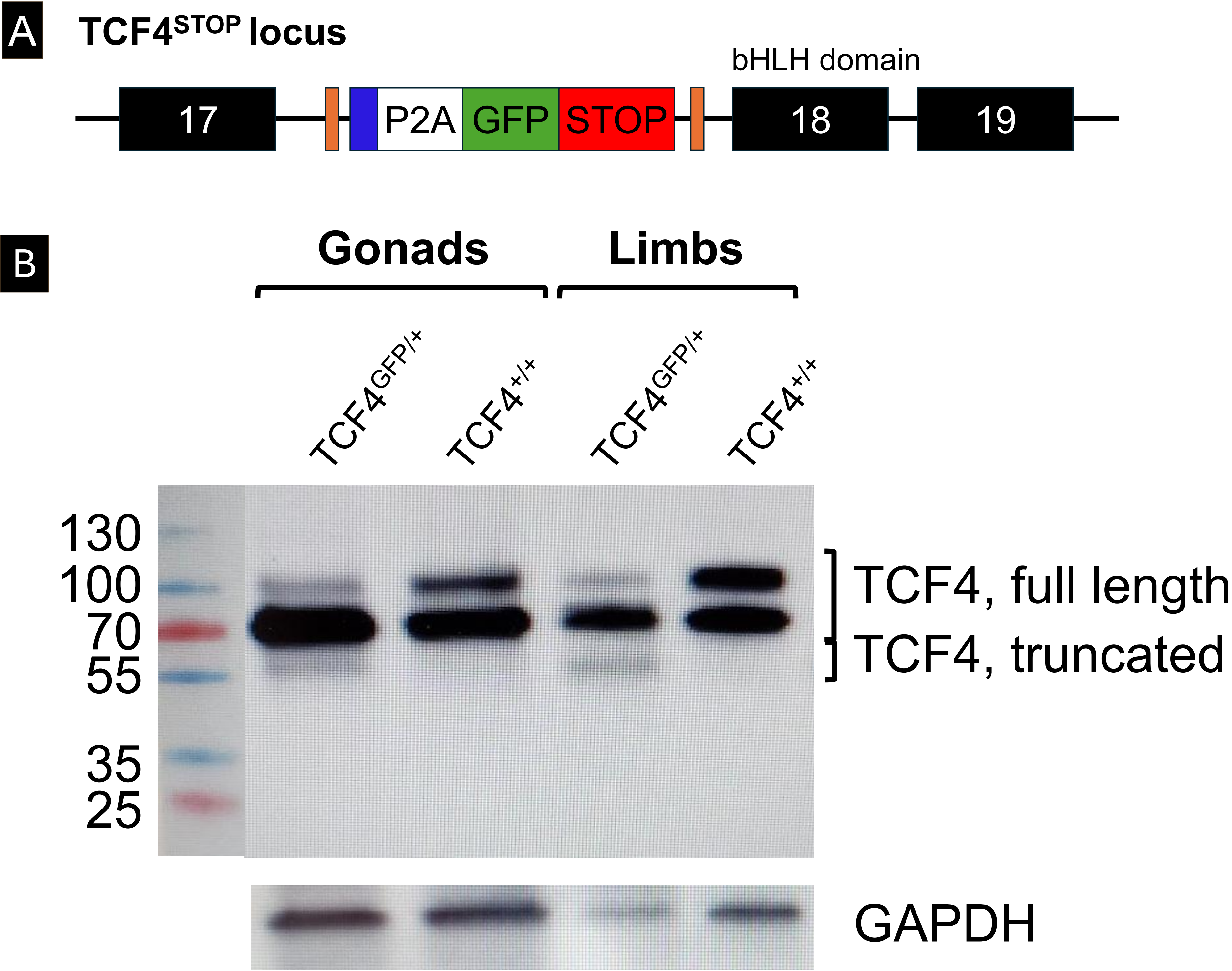

Supplemental Figure 3

A

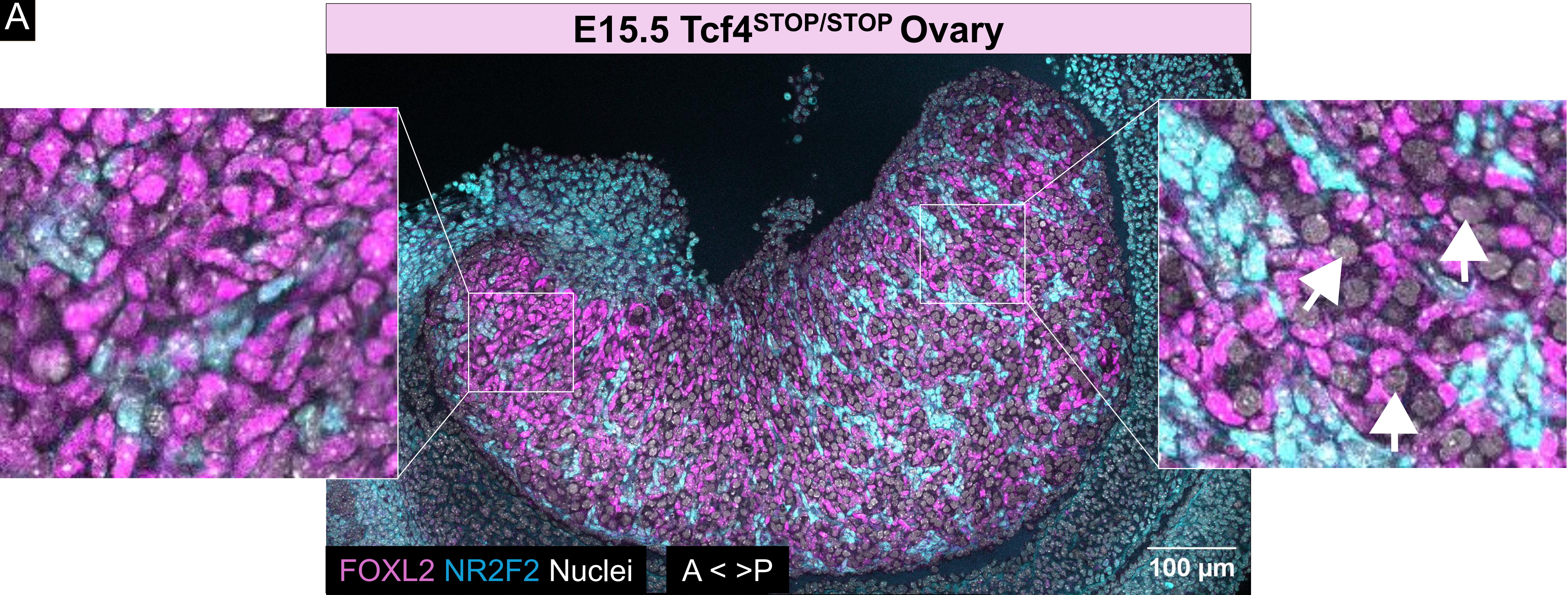

B

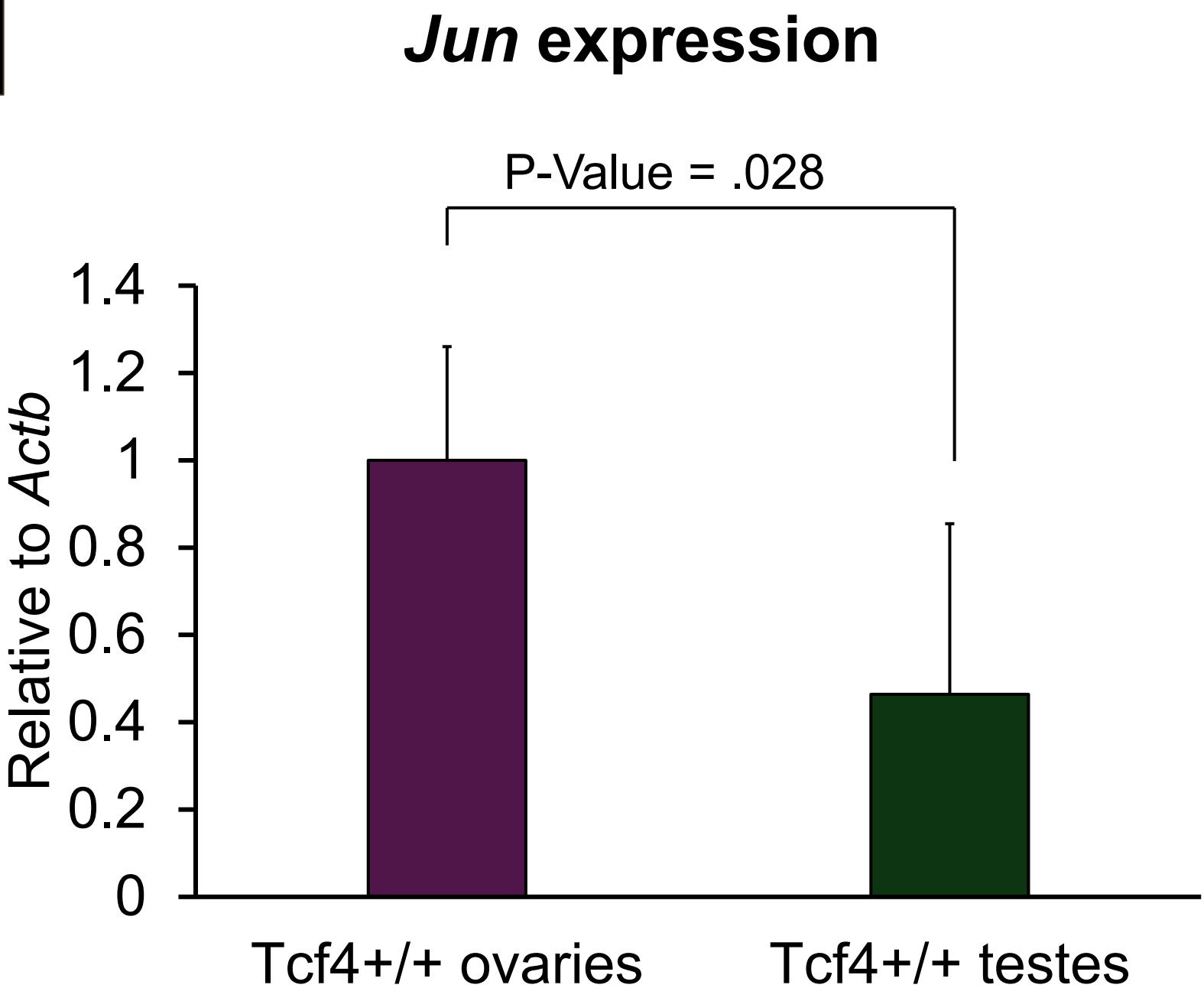

Supplement: Supplement 5 — Figure S1. Expression of TCF4 is maintained in gonadal granulosa and interstitial cells through late gestation. A) At E12.5, TCF4 co-localizes with VCAM- and NR2F2- expressing interstitial cells in XY gonads. B) At E18.5, TCF4 remains restricted to the testicular interstitial cells and absent in SOX9-expressing Sertoli cells. C) At E18.5, TCF4 is broadly expressed across both granulosa and interstitial cell lineages in the oary. Scale bar represents 100 μm. Microscopy images are stitched together from individual panels. Figure S2. The TcfSTOP allele is unable to bind DNA. A) The TcfSTOP allele introduces a premature STOP codon upstream of the bHLH domain in exon 18. B) Western blot analysis shows that TcfSTOP/+ mice express a truncated form of TCF4 without the bHLH domain that is absent in Tcf+/+ wildtype littermates. Figure S3. Minor defects in TcfSTOP/STOP mutant ovaries. A) One TcfSTOP/STOP mutant ovary displayed defects in patterning, as germ cells were present in the posterior portion of the ovary (white arrowheads) but were absent in the anterior portion. B) The transcription factor Jun is expressed in Tcf+/+ wildtype testes at about half the level of Tcf+/+ wildtype ovaries. [file media-5.pdf]
