# Supplementary material for: The basic helix-loop-helix transcription factor TCF4 recruits the Mediator Complex to activate gonadal genes and drive ovarian development
Source: bioRxiv. 2025 Mar 4:2025.02.28.640455. Preprint. [Version 1] doi: 10.1101/2025.02.28.640455 (PMC11908221; doi:10.1101/2025.02.28.640455)

Supplemental Figure 1. TCF4 localization in the developing gonad. A) TCF4 co-localizes with interstitial markers (VCAM and NR2F2) in the XY gonad. B) By E18.5, TCF4 is enriched in the testis interstitium. TRA98 marks germ cells and SOX9 marks Sertoli cells. C) By E18.5, TCF4 localizes with granulosa cells and interstitial cells in the ovary. NR5A2 marks granulosa cells and ACTA2 marks smooth muscle actin. Scale bar is 100µm.

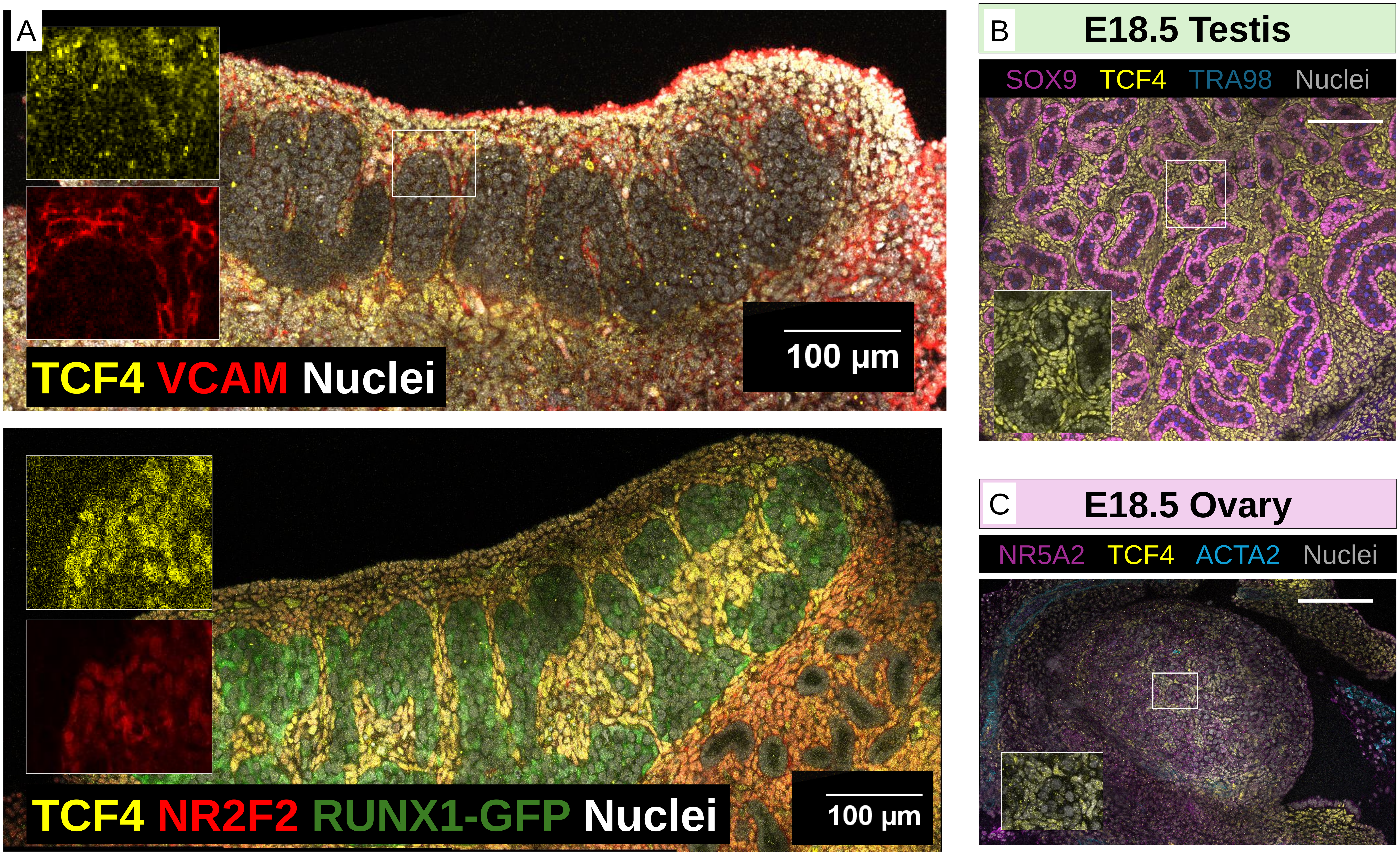

Supplemental Figure 2. Truncated TCF4 in TCF4$^{STOP/+}$ mutant gonads and limbs. The TCF4^{STOP} mutation leads to a truncated form of TCF4 that is unable to bind DNA. 20 µg protein per lane.

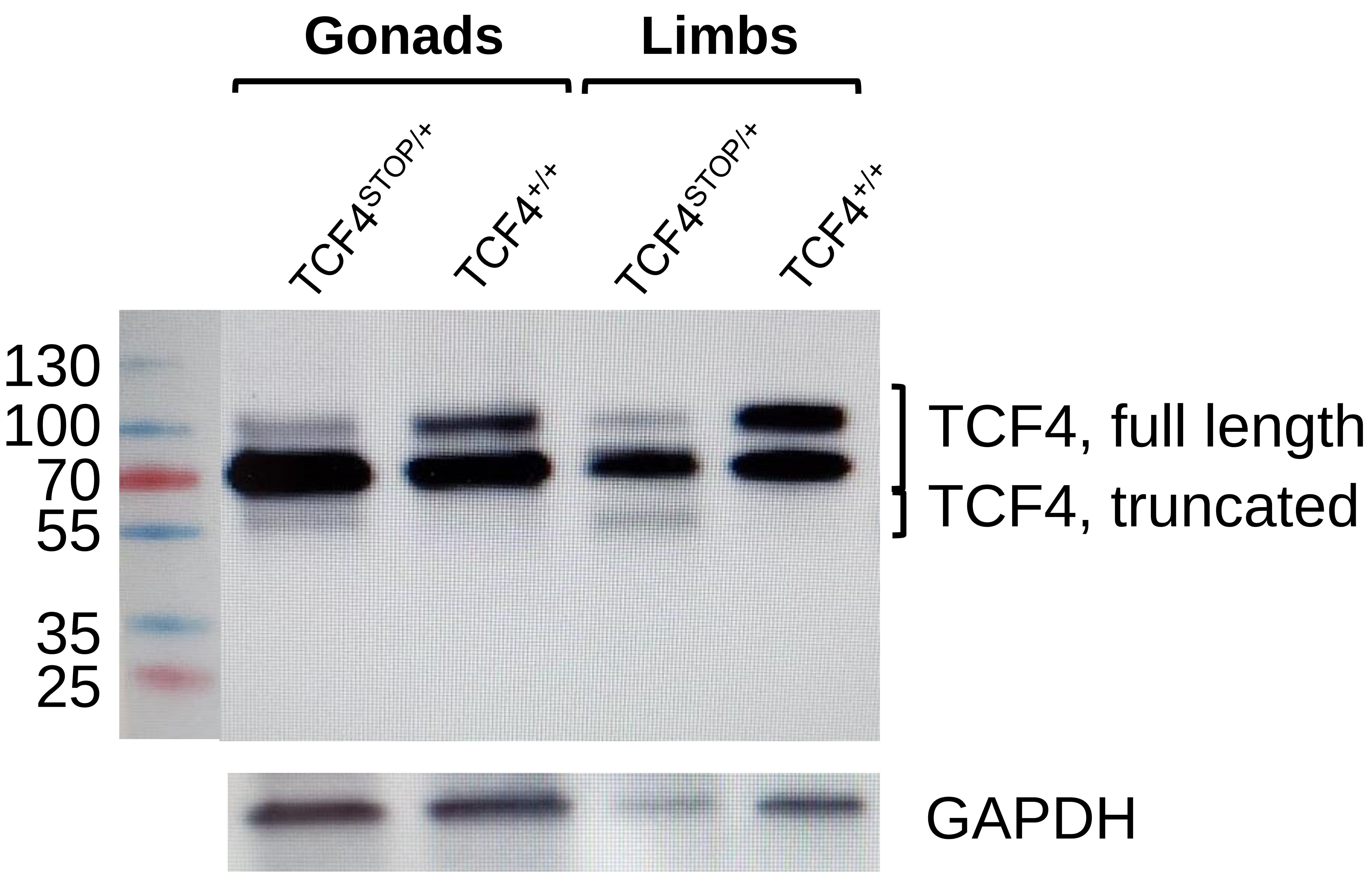

Supplemental Figure 3. TCF4$^{STOP/STOP}$ mutant ovary. TCF4$^{STOP/STOP}$ mutant mice developed gonads, but some mutants had pockets of ovarian cells without germ cells, suggesting disruptions to ovary patterning.

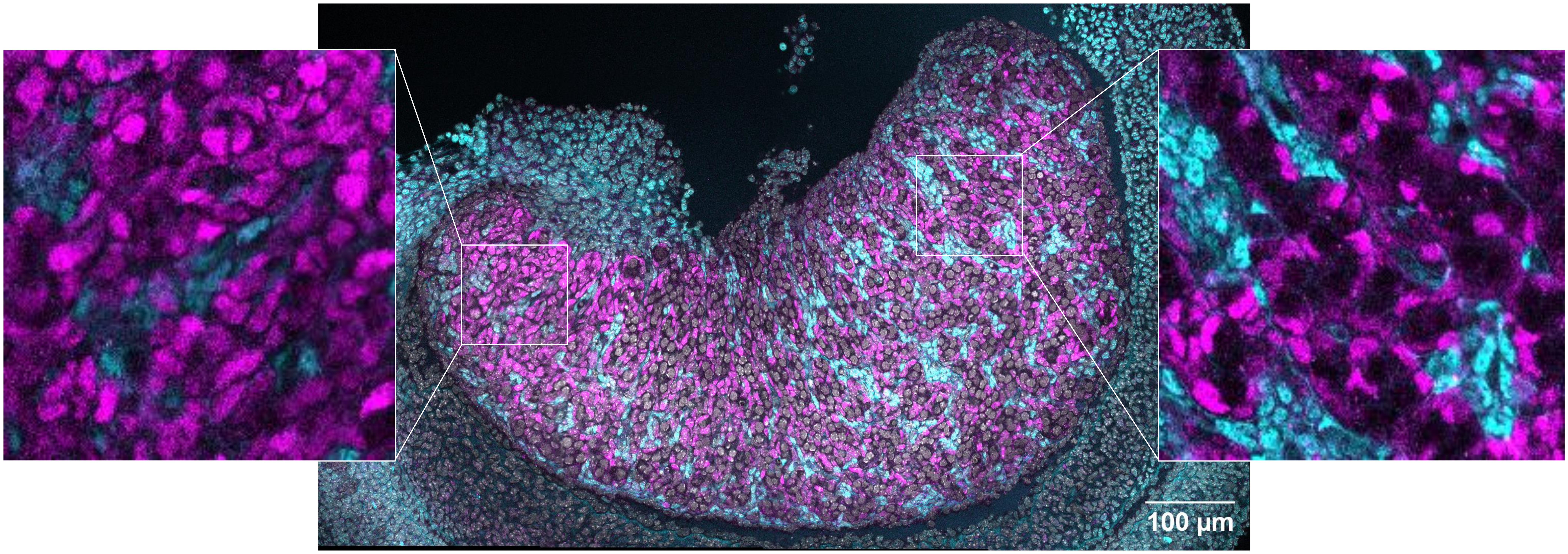

Supplement: Supplementary file 1 [file media-4.pdf]
